# A novel heat treatment protocol for human milk

**DOI:** 10.3389/fped.2022.990871

**Published:** 2022-10-18

**Authors:** Olimpia A. Manzardo, Luisa J. Toll, Katharina Müller, Erika Nickel, Daniel Jonas, Jana Baumgartner, Folker Wenzel, Daniel Klotz

**Affiliations:** ^1^ Department of Neonatology, Center for Pediatrics, Medical Center, Faculty of Medicine, University of Freiburg, Freiburg, Germany; ^2^ Faculty of Medical and Life Sciences, Furtwangen University, Furtwangen, Germany; ^3^ Institute for Infection Prevention and Hospital Epidemiology, University of Freiburg, Freiburg, Germany

**Keywords:** bacteria, human milk, pasteurisation, protein, premature infant

## Abstract

Human milk (HM) is the recommended nutrition for premature infants, but it may require processing to ensure microbial safety. The current standard is Holder pasteurisation (HoP), i.e. heating milk at 62.5 ± 0.5°C for 30 min, which eliminates bacteria but destroys heat labile bioactive HM components. We aimed to test an alternative thermal method, high-temperature short-time (HTST) pasteurisation using a modified Holder pasteurisation platform as this method has shown to preserve proteins in experimental HM flow pasteurisers. We analysed the ability of this batch process to eliminate bacterial species and to retain alkaline phosphatase, secretory immunoglobulin A and lactoferrin in HM. HTST at 81°C/5 s was as effective as HoP in bacterial count reduction while the retention of bioactive components was only improved at 62°C/5 s as compared to 72°C/5 s and HoP. HTST is a promising alternative to HoP but an optimal temperature-time combination needs to be determined for each technical platform separately.

## Introduction

Human milk (HM) is the recommended enteral nutrition for infants (1–3). In the context of extreme prematurity, microbial colonisation of HM represents a potential health hazard to the premature infant. Therefore, pasteurisation of HM for extremely premature infants is frequently performed (4). Holder pasteurisation (HoP) is the current gold standard of thermal HM treatment: a low-temperature, long-time pasteurisation method that heats milk to a plateau temperature of 62.5 ± 0.5°C for a holding time of 30 min. Given that every milk particle is heated homogenously, this treatment results in the inactivation of viruses, namely Cytomegalovirus (CMV) and allows a 5-log_10_ reduction in bacterial content, as required by guidelines of most human milk banks worldwide (5, 6).

The adverse, heat dependent effects of HoP on the bioactive components of HM are well described. These effects collectively reduce the beneficial potential of HM for premature infants and might ultimately affect their clinical outcome (7). Therefore, alternative methods are being explored to reduce the heat exposure of bioactive components, while still guaranteeing microbial safety (8). Different approaches are under study, e.g., reducing heat exposure through modulation of the time-temperature curve, such as the high-temperature short-time (HTST) pasteurisation method. By increasing the plateau temperature and drastically decreasing the ramp, plateau and cooling time, the overall heat exposure is reduced and protein retention in HM is improved (8). Many different types of experimental pasteurisers using either batch or flow-through techniques have been evaluated for their efficacy to provide microbial safety in HM. Unfortunately, none of these prototypes are currently commercially available for clinical use (6).

Therefore, we adapted an available HTST treatment device that was originally designed for CMV inactivation in human milk but has shown to be inefficient in bacterial count reduction at the default setting of 62°C for 5 s (9). Our primary aim was to demonstrate that this short-time treatment process is as effective as HoP in reducing bacterial counts in artificially incubated HM using adequate time-temperature combinations, thus qualifying as pasteurisation process. Subsequently, we hypothesized that the retention rate of bioactive human milk components would be increased after HTST treatment compared to HoP.

## Materials and methods

### Milk sampling and preparation

Human milk that exceeded the nutritional needs of her child and could not be used as donor human milk was donated by the mother of a premature infant treated in our neonatal intensive care unit (NICU) after obtaining written informed consent from the donor and being granted approval from the Ethics Committee of the University of Freiburg. Maternal milk was mechanically expressed with milk pumps (Medela, Baar, CH) into precooled infant feeding bottles (Beldico, Marche-en-Famenne, BEL). The samples were immediately chilled at 4°C and frozen at −20°C within 48 h after expression until further processing. We collected a total of 2400 ml of donated milk.

**Figure 1:**
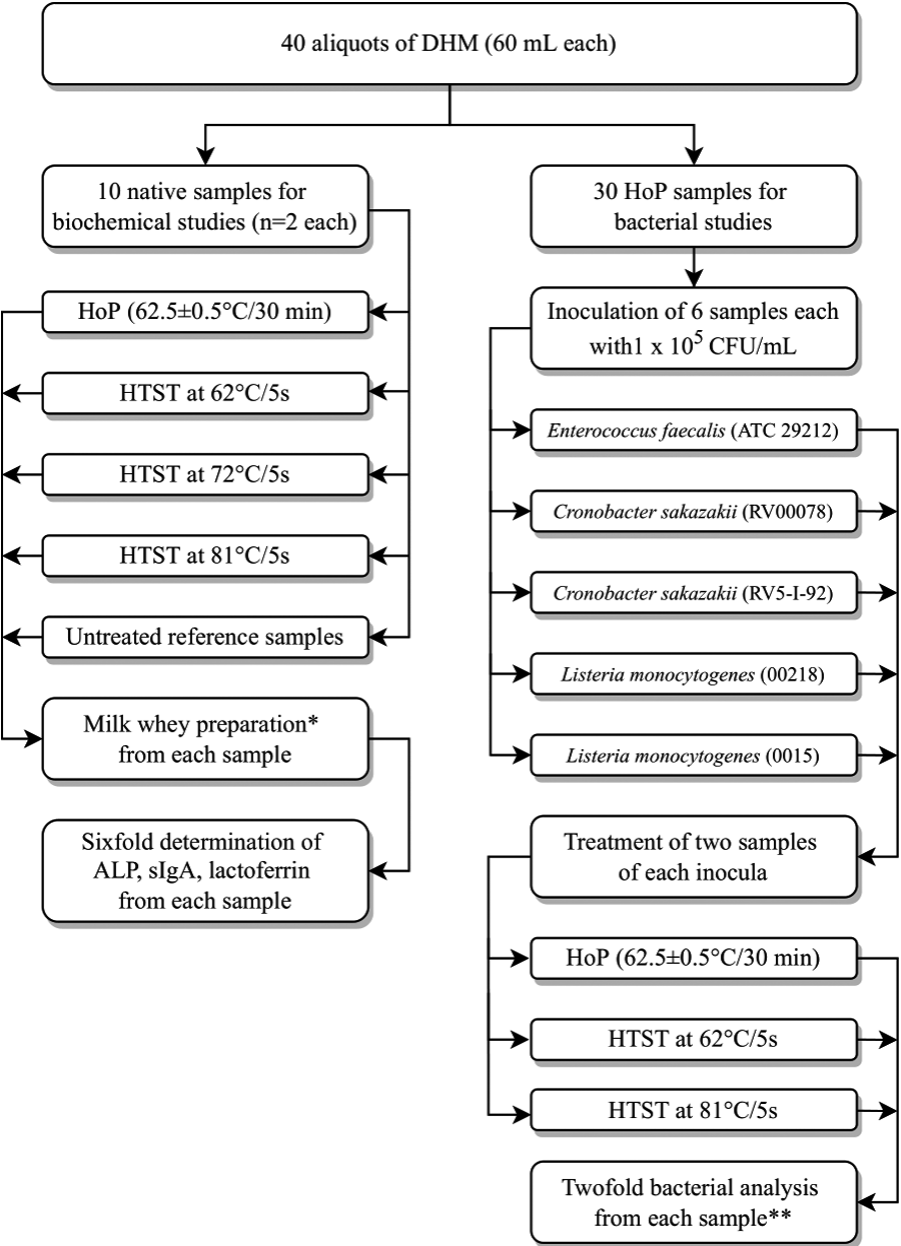
Study flow chart: processing of human milk probes. ALP = alkaline phosphatase; CFU = colony forming units; DHM = donor human milk, HoP = Holder pasteurisation; HTST = high temperature short time treatment; sIgA = secretory immunoglobulin A. *Preparation of milk whey: From samples 10 ml aliquots were centrifuged at 600 g for 10 min, the resulting aqueous layer was centrifuged again at 600 g for 10 min and the resulting whey supernatant was centrifuged at 2,500 g for 10 min and the resultant supernatant was then filtrated with a 0.45 μm with syringe filter. **Lower limit of detection < 10 cfu/ml, inoculated but not heat-treated aliquots were also analyzed.

For further processing the samples were thawed at 4°C overnight, subsequently pooled and divided into 40 aliquots à 60 ml each. The process flow chart is given in Figure 1. At any stage samples were refrigerated at 4°C when not being processed.

### Bacterial inoculation

For bacterial studies 30 milk samples were subjected to HoP as detailed below in order to eliminate any naturally colonizing milk bacteria before artificially inoculating the samples. Bacteria for artificial inoculation of HM were cultured on Columbia blood agar at 36 ± 0.5°C overnight (Thermo Fisher, Waltham, US). Thereafter colonies were suspended in 0.9% sodium chloride and adjusted to the desired concentration of 10^8^ CFU/ml using the optical density method with a turbidity metre at 620 nm (Dade Behring, Sacramento, CA, US). Bacterial concentrations were confirmed by plating dilutions onto Columbia blood agar and counting colonies subsequent to overnight incubation at 36°C. Concentration of the bacterial suspension was 1 × 10^8^ CFU/ml, of which 60 µ were added to a 60 ml HM sample resulting in an inoculation dose of 1 × 10^5^ CFU/ml, since pasteurization systems in general are designed to provide a 5 log reduction of a microbial load. According to recommendations about the evaluation of new pasteurization techniques six aliquots of HoP pasteurised milk each were then inoculated with either *Enterococcus faecalis* (ATC 29212) or with the clinical isolates of *Cronobacter sakazakii* (RV00078), *Cronobacter sakazakii* (RV5-I-92), *Listeria monocytogenes* (00218) and *Listeria monocytogenes* (0015) and processed immediately thereafter (6).

Those six aliquots for each inoculum were treated as follows: Holder pasteurisation (*n* = 2) at 62.5 ± 0.5°C for 30 min, HTST treatment at 62°C for 5 s (*n* = 2) or HTST at 81°C for 5 s (*n* = 2) as described below.

### Heat treatment

For the HTST treatment, we used a custom-made adaption of a previously described device for CMV inactivation (Virex II, Lauf, Tuebingen, Germany) (5). Aliquots were individually placed into a rotating glass flask and the resulting milk film was heated by a default setting to a plateau temperature of 62°C and held at this temperature for 5 s before being rapidly cooled with cold water to approximately 32°C within the device. The probes were then placed into an ice bath and chilled to 4°C and immediately processed thereafter. For the purpose of this study additional adjustable time-temperature combinations were added to the device to achieve plateau temperatures of 72°C (for biochemical analysis only, see below) or of 81°C for a respective plateau time of 5 s.

Holder pasteurisation was performed using a portable dry tempering aluminium alloy device (Clinitherm Pasteur, MedCare Visions, Germany) as previously described, within the premises of the microbial laboratory of the Institute for Infection Prevention and Hospital Epidemiology (10). After pasteurisation at 62.5 ± 0.5°C for 30 min, samples were chilled to 4°C in an ice bath and immediately processed thereafter.

The resulting time-temperature curves during Holder pasteurisation were measured in reference bottles as per manufacturers' instruction (MedCare Visions). Additionally, for the purpose of this study, we measured time-temperature curves using temperature probes (176T4, Testo, Neustadt, Germany) in further reference bottles.

**Figure 2:**
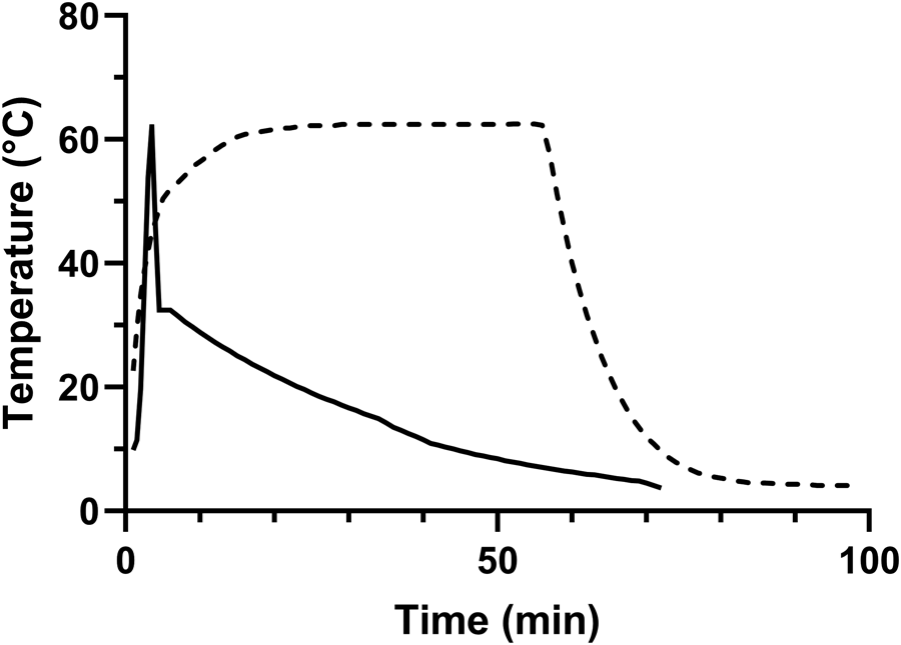
Exemplary time-temperature curve of the pasteurisation processes. Dashed line = Holder pasteurisation; Straight line = high temperature short time (HTST) treatment.

The time-temperature curve of the HTST treatment (Virex) was measured with a default spring mounted sensor that was constantly in contact with the heated fluid layer. Additionally, the plateau-temperature in this adapted model was measured *via* a second channel within the spring mounted sensor and connected to an external thermometer (Logo Siemens, Berlin, Germany) that was purposely designed for this study. Data of the pasteurisation processes were digitally recorded using a purpose-made software and externally stored *via* port (MedCare Visions), removable memory hard drive (Virex) as provided by the manufacturers and an external datalogger (Testo) (Figure 2).

HM samples were stored at 4°C at all times in between further treatments.

### Microbial analysis

Bacterial cultures for microbial analysis after pasteurisation were performed inoculating Columbia blood agar (Thermo Fisher, Waltham, USA) with 100 µl of milk from each sample and incubated at 36 ± 0.5°C and 7.5% carbon dioxide in a Heracell incubator (Thermo Fisher). Colony forming units per millilitre (CFU/ml) were determined visually after 24 h and 48 h. Identity of bacterial species was confirmed by use of a MALDI-TOF (Bruker Daltonik, Bremen, Germany).

### Biochemical analysis

The flow chart of biochemical analysis is given in Figure 1. Samples were Holder pasteurised or received HTST treatment at 62°C, 72°C or at 81°C for 5 s as aforementioned. One reference sample remained untreated. The samples were processed for biochemical analysis immediately after the heat treatments. We prepared 3 × 10 ml aliquots from each pasteurised sample and the native reference sample (Greiner Bio oneFalcon test tube, BD, New Jersey, USA) and centrifuged the aliquots at 600 g for 10 min. The resulting aqueous layer was centrifuged again at 600 g for 10 min and the supernatant was then centrifuged at 2500 g for 10 min. The resulting supernatant was filtered with a syringe filter 0.45 µm (Pall, NY, USA) and stored frozen for less than 72 h at −20°C until biochemical analysis.

Frozen filtered aliquots were thawed at room temperature for biochemical analysis. We performed functional assays to determine alkaline phosphatase (ALP) activity within the samples (ALP Activity Assay Kit, Biovision, Milpitas, US). Concentrations of lactoferrin (Lf) and secretory immunoglobulin A (sIgA) were measured *via* enzyme-linked immunosorbent assay (ELISA) kits as per manufacturers direction (Lf: Abcam, Cambridge, UK; sIgA: Demeditec Diagnostics, Kiel, Germany).

### Data analysis

We performed bacterial cultures in duplicates for each tested inoculum before and after pasteurisation. Pasteurisation was also performed in duplicates for each inoculum and each treatment process. We converted bacterial colony counts to log_10_ values for statistical analysis and performed a one-sample *t*-test against a hypothetical value of zero to compare the mean reduction of bacterial counts by HTST treatments with the reduction observed after Holder pasteurisation.

Whey proteins were measured as duplicates from each of the three aliquots of the native reference sample and the heat-treated samples. Mean values were compared using ANOVA, *t*-test or sign-rank test where appropriate (GraphPadPrism V8, GraphPad, US). We considered *p*-values of <0.05 to be statistically signiﬁcant.

## Results

### Microbial analysis

Final inoculation doses of the milk samples are given in Table 1. Milk samples after Holder pasteurisation did not reveal any bacterial growth after cultivation for 24 and 48 h, resulting in a reduction of at least 4.76 orders of magnitude for each bacterial species inoculated, with the exception of one batch with a growth of *Cronobacter sakazakii*.

**Table 1:** Bacterial concentrations before and after heat treatment.

	Unpasteurised	HoP 62.5°C/30 min		HTST 62°C/5 s		HTST 81°C/5 s	
Inoculated species	CFU/ml	CFU/ml	Log reduction	CFU/ml	Log reduction	CFU/ml	Log reduction
*Enterococcus faecalis* (ATCC29212)	5.8 × 10^4^	0*	4.76	1 × 10^4^	0.76	2.7 × 10^1^	3.32
*Cronobacter sakazakii* (RV00078)	1.4 × 10^5^	2.5 x10^1^	4.75	1.6 × 10^3^	1.93	0*	5.15
*Cronobacter sakazakii* (RV5-I-92)	2.1 × 10^5^	0*	5.32	1 × 10^4^	1.32	0*	5.32
*Listeria monocytogenes* (00,218)	1.8 × 10^5^	0*	5.26	1 × 10^4^	1.26	5	5.26
*Listeria monocytogenes* (0015)	1.1 × 10^5^	0*	5.04	1 × 10^4^	1.04	0*	5.04

* lower limit of detection = 10 CFU/ml. Of each species six aliquots were inoculated (*n* = 2 for HoP, 62°C and 81°C respectively).

CFU, colony forming units; HoP, Holder pasteurisation; HTST, high temperature short time treatment.

In contrast, after HTST treatment at 62°C/5 s we retrieved viable cultures for every tested species (Table 1). HTST treatment at 81°C/5 s resulted in a > 5 log_10_ reduction of all inoculated bacteria with the exception of *E. faecalis* (reduction of 3.32 orders of magnitude)*.* There was no difference in the bacterial counts after both HoP and HTST treatment at 81°C/5 s when tested to a hypothetical value of 0 (HoP, *p* = 0.37 and HTST, *p* = 0.29 respectively). In contrast, HTST treatment at 62°C/5 s was not effective in bacterial count reduction when tested to a hypothetical value of 0 (*p* = 0.008), i.e., HoP.

### Biochemical analysis

In the untreated milk samples mean lactoferrin concentration and standard deviation (SD) were 9.27 ± 1.37 g/L, 0.27 ± 0.05 g/L for sIgA and mean (SD) ALP activity was 0.33 ± 0.03 mU/ml. ALP activity, lactoferrin and sIgA concentrations after heat treatment are given in Table 2.

**Table 2:** Protein concentrations before and after heat treatment.

	ALP (mU/ml)	Lactoferrin (g/l)	sIgA (g/l)
Unpasteurised	0.33 ± 0.03	9.27 ± 1.37.	0.27 ± 0.05
HoP 62.5°C/30 min	0*	2.04 ± 0.31	0.21 ± 0.05
HTST 62°C/5 s	0.09 ± 0	4.93 ± 0.70	0.22 ± 0.10
HTST 72°C/5 s	0*	2.48 ± 0.46	0.05 ± 0.03
HTST 81°C/5 s	0*	2.01 ± 0.65	0**

Shown are mean ± standard deviation from 6 measurement of each parameter for each treatment.

* lower limit of detection = 0.005 μU/well.

** lower limit of detection = 0,06 µg/ml.

HoP, Holder pasteurisation; HTST, high temperature short time treatment ALP, alkaline phosphatase; sIgA, secretory immunoglobulin A.

**Figure 3:**
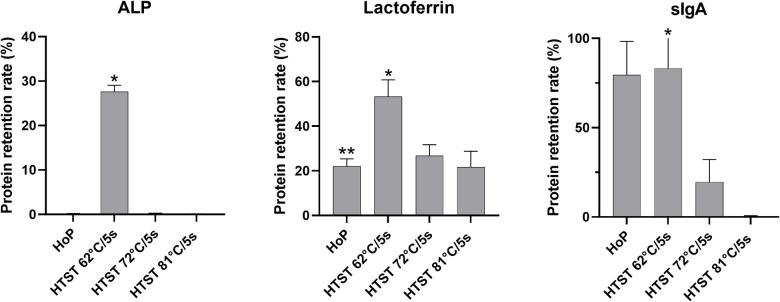
Protein retention rates after thermal treatment from 6 measurement of each parameter for each treatment. ALP, alkaline phosphatase; sIgA, secretory immunoglobulin A; HoP, Holder pasteurisation; HTST, high-temperature short-time treatment *p < 0.001(HTST 62°C vs. HTST 72°C or HTST 81°C), **p = 0.31(HoP vs. HTST 72°C or HTST 81°C).

Retention of ALP activity was not detectable after HoP or HTST at 72°C or 81°C respectively (*p* = 0.84). In contrast, HTST treatment at 62°C/5 s resulted in a substantial ALP retention rate compared to HoP and HTST treatment at 72°C or 81°C (all *p* < 0.001, Figure 3).

Lactoferrin concentration was substantially conserved after HTST treatment at 62°C/5 s compared to HTST treatment at 72°C/5 s or 81°C/5 s (both *p* < 0.001) or HoP (*p* = 0.03, Figure 3), while there was no difference in the lactoferrin concentration after HoP and HTST treatment at 72°C/5 s or 81°C/5 s (*p* = 0.31).

Secretory IgA retention rate was 79.5% after HoP and 83.2% after HTST treatment at 62°C/5 s (*p* = 0.85). In contrast, sIgA concentrations were significantly reduced after both HTST treatment at 72°C/5 s and at 81°C/5 s compared to both HoP and HTST treatment at 62°C/5 s (all *p* < 0.001).

## Discussion

In our study, we demonstrated that a 5 s short-time heat treatment of human milk may be as effective as Holder pasteurisation in bacterial count reduction for most tested bacterial species but only when increasing holding temperatures to 81°C. However, increasing holding temperatures resulted in reduced protein retention rates compared to the classical Holder method, i.e., heating milk at 62.5 ± 0.5°C for 30 min.

Our results concerning the antibacterial efficacy of HTST treatments of human milk are in line with previous reports. Bacterial count reduction using HTST was found to be as efficient as HoP in naturally colonized human milk, using a range of holding times from 1 to 25 s and plateau temperatures from 62.5°C to 87°C (11–14). Challenge tests on artificially inoculated human milk confirmed the antibacterial efficacy of different HTST prototypes (9, 15–17). In general, bacterial genera and species used in challenge tests or found in naturally colonized milk vary greatly which may have an impact on the observed antibacterial efficacy of different HTST platforms and time-temperature combinations in question. Furthermore, diverging heat susceptibilities of different strains of some species have been observed resulting in an even less predictable outcome of thermal HM treatments (9). Invariably, some bacterial species, i.e., *Enterococcus faecalis* and *Bacillus cereus*, could be consistently recovered after both HTST and HoP treatment, highlighting their inherent thermoduric heat resistance or their spore forming abilities (9, 14).

From a clinical point of view, however, defining a safe HM microbiome remains elusive and many different thresholds pertaining to the spectrum and concentration of HM bacteria have been considered as being acceptable for feeding premature infants (4, 18, 19). In conclusion, our findings suggest that, as previously published, HTST at 62°C/5 s or 75°C/5 s could be considered being sufficient to reduce bacterial concentrations, especially of those bacteria that can be regularly found in raw HM and that are relevant in a clinical setting, to a threshold that may be considered safe by clinicians (9).

Using our modified Holder device for HTST treatment we were able to demonstrate increased retention rates of bioactive HM components. As expected, ALP was inactivated after HOP and HTST at 71°C and 81°C whereas it was mostly preserved at 62°C, in line with our previous observation (13). Complete ALP inactivation has been defined as prerequisite for the definition of the term *pasteurisation* of milk of animal origin in some legislations and this has also been applied to HM by some researchers (20). This definition, however, may not be applicable when intending to retain as many bioactive milk components as possible when treating human milk for premature infants. Lactoferrin retention rates were substantially improved at 62°C compared to HoP, while we did not observe a similarly increased retention at 72°C as opposed to previous reports that tested tubular flow pasteurisers (21, 22). Secretory IgA retention rates were increased after HTST at 62°C compared to HoP, in line with our previous observations (13). The retention rate of sIgA at 71°C for 5.7 s was previously given at 68%–83% unlike our results (18% retention rate), again using a tubular flow pasteuriser (11, 15). Secretory IgA was significantly reduced or could not be retrieved as in our case when increasing the plateau temperature above 80°C (11, 21).

Nonetheless, the above-mentioned results of bacterial and biochemical studies were obtained using a range of different technical platforms, i.e., plate heat exchanger, continuous tubular heat exchanger and oscillating batch systems. This makes comparisons between studies that are exclusively based on applied time-temperature protocols difficult. Inherently prolonged cooling times of milk treated in batch devices compared to more rapid cooling times of tubular flow-based devices may be responsible for this observed difference. In general, batch pasteurisers loaded with single donor specimens as tested in our study may be preferred to flow-based pasteurisers in a human milk bank setting. Firstly, for several reasons, human milk may not be pooled from multiple donors and small and variable volumes of human milk may be provided per lactating mother per day. Therefore, small volumes of HM from different donors would need to be processed successively. In this scenario, processing of HM would be interrupted by multiple cleaning and disinfection cycles per work day, thus limiting the efficiency of flow-based devices. Secondly, device specific loss rate of human milk during processing may be higher in flow-based pasteurisers compared to the Holder approach. This is relevant in a setting with limited supply of human milk where very small quantities of milk are sufficient to cover the enteral nutrition needs of a premature infant. However, the above-mentioned differences in cooling times between the two different technical platforms must be considered and comparative assessments pertaining to efficacy and efficiency of different technical HTST platforms are lacking. In conclusion, HTST using the present device did increase protein retention when applying 62°C but results were inconsistent regarding higher temperature settings.

Our study has some limitations. We analysed a limited set of bioactive HM components, even if previous use of these HM proteins allows to compare protein retention rates. Future studies should aim at increasing the coverage of the tested HM components. For feasibility reasons we limited our observations to three time-temperature protocols, nonetheless, we applied a rigorous protocol as recommended for testing the effects of processing on donor human milk (23). More time-temperature variations should be evaluated to find an optimal HTST protocol able to sufficiently reduce bacterial counts, while still maintaining a satisfactory concentration of immunological and nutritive human milk components. In addition, given the inefficiency of thermal treatments towards spores and the persistent reduction of thermosensitive milk proteins, other non-thermal methods such as hyperbaric pressure and UV-C pasteurisation may also be evaluated in this context (6).

Due to the detrimental effects of HoP, colonised HM may be discarded instead subjected to HoP and substituted by formula (4). Alternatives to HoP are therefore urgently needed. We could show that HTST batch pasteurisation is a promising technique for HM processing, with the potential of improving the retention of bioactive components. However, to achieve an antibacterial efficacy comparable to HoP further studies are required to determine the optimal heat exposure.

## Data Availability

The original contributions presented in the study are included in the article/Supplementary Material, further inquiries can be directed to the corresponding author/s.
